# Analysis of top box office film poster marketing scheme based on data mining and deep learning in the context of film marketing

**DOI:** 10.1371/journal.pone.0280848

**Published:** 2023-01-26

**Authors:** Shuyuan Yang

**Affiliations:** School of film, Xiamen University, Xiamen City, China; Sejong University, REPUBLIC OF KOREA

## Abstract

With the development of science and technology and the continuous changes of social environment, the development prospect of traditional cinema is worrying. This work aims to improve the publicity effect of movie posters and optimize the marketing efficiency of movie posters and promote the development of film and television industry. First, the design concept of high grossing movie posters is discussed. Then, the concept of movie poster analysis based on Deep Learning (DL) technology is analyzed under Big Data Technology. Finally, a movie poster analysis model is designed based on Convolutional Neural Network (CNN) technology under DL and is evaluated. The results demonstrate that the learning curve of the CNN model reported here is the best in the evaluation of model performance in movie poster analysis. Besides, the learning rate of the model is basically stable when the number of iterations is about 500. The final loss value is around 0.5. Meanwhile, the accuracy rate of the model is also stable at the number of iterations of about 500, and the accuracy rate of the model is around 0.9. In addition, the recognition accuracy of the model designed here in movie poster classification recognition is generally between 60% and 85% in performing theme, style, composition, color scheme, set, and product recognition of movie posters. Moreover, the evaluation of the model in the movie poster style composition suggests that the style composition of movie poster production dramatically varies in different films, in which movie posters focus most on movie product, style, and theme. Compared with other models, the performance of this model is more outstanding in all aspects, which shows that this work has achieved a great technical breakthrough. This work provides a reference for the optimization of the design method of movie posters and contributes to the development of the movie industry.

## Introduction

With the progress of science and technology, many information communication technologies have emerged, and film and television technology has become the mainstream information communication technology model. Currently, posters are one of the most dominant art forms, especially in the film and television industries [1]. Film posters play a crucial role in film development and advertising. However, the traditional film and television technology has been unable to meet the needs of the market in the current state of scientific and technological development and the continuous transformation of the social environment. Therefore, the continued development of the film and television industry is essential to optimize the design means of the film and television industry. It is necessary to design high-quality film posters through the most advanced scientific and technological means to strengthen film publicity and promote the development of the film industry [2]. Moreover, with the continuous development of science and technology, there is nothing that can currently adapt to this need than machine learning technology. Deep Learning (DL) is an advanced machine learning method that can undertake various tasks of calculation, classification, and prediction. Therefore, optimizing the design of movie posters through DL techniques is an innovative and long-term concept [3]. In recent years, the film industry has shown a state of inactivity due to the social environment. Therefore, it is imperative to study the future development of the film industry through advanced science and technology to improve the accuracy of predicting the development of the film industry and to promote the development of the film industry. To this end, this work studies the development of the movie industry through the integration of Big Data Technology (BDT) and DL technology. Although the application of DL in movie poster design is not mature enough, many studies provide technical support.

Dewi and Khristianto (2022) reported that movie posters are a vivid record of the development of movie art. Some old posters can remind people of the classic movies they used to be familiar with, which are cordial and warm and full of the humanistic feelings and aesthetic views of that era. Movie posters are closely related to the works, and their design has special restrictions and requirements. Therefore, it is worth analyzing how to design an attractive a good movie poster [4]. Suk and Kim (2021) pointed out that the movie poster is a window for the public to obtain movie information and the embodiment of the designer’s aesthetic pursuit of cultural values. Poster design must conform to the development of the times and fully use new means and methods in expression to meet the requirements of the times. The aesthetic function is a prerequisite for movie posters to transmit information and the aesthetic requirement of the times for movie poster design. The design of movie posters in the new era, primarily the poster works with positive artistic and social significance, will undoubtedly show people’s inner spirit and freedom of mind at a high level [5]. Wi et al. (2020) pointed out that movie posters convey information on the one hand. Besides, they are independent art that can exist alone as a work of art. Therefore, touching people’s emotions through images is a prominent feature of movie posters. It shows that the organic unity of the content and form of the movie poster image is the key to realize the function of the movie poster [6]. Tulbure et al. (2022) revealed that accurate image recognition is of great research significance, and image recognition technology plays a vital part in medicine, aerospace, military, industry and agriculture, and many other fields. Most of the current image recognition methods use artificial extraction of features, which is time-consuming and labor-intensive, and difficult. is a kind of Unsupervised Learning, which does not require the label value of the sample and manual participation during the learning process to extract features. In recent years, the application of DL to image recognition has become a research hotspot in the field of image recognition and achieved good results [7]. Alfian et al. (2019) used customers’ browsing history and digital signage (DS) transaction data as input for decision-making to extract customer behavior patterns. First, the author developed DS and installed it in different locations so that customers can have the experience of browsing and purchasing products. Second, real-time data processing systems collected customers’ browsing history and transaction data. In addition, the authors utilized association rules to extract useful information from customer behavior, so managers can use it to effectively improve service quality [8].

The structure of this work is as the follows. Firstly, the basic content of movie poster is discussed, including the discussion on the design method, concept, and meaning of movie poster. Then, the application concept of DL technology in movie poster design under BDT is introduced. Finally, the DL movie poster analysis model is designed, and the model is evaluated. The innovation of this work is to provide a new technical approach to the analysis and design of movie posters and realize the intelligence of movie poster design analysis. Therefore, the specific contributions of this work are three-fold. First, the technical update of this work provides a technical reference for the optimization of DL technology. Secondly, the application of DL technology in film and television industry studied here provides a reference for the future expansion of the application scope of DL technology. Third, this work contributes to the intelligent transformation as well as the far-reaching development of film and television industry.

## DL-based film poster analysis under BDT

### Film poster

1) The origin of film posters

Since its inception, consumers have loved movies as a form of art and entertainment. The film market has been in vigorous development. The connotation of film posters is film advertising. Initially, film posters were mainly used for film promotion, which was the product of the social environment and the development of the times under the creation of films [9]. Film posters mainly use creative means of painting, writing, photography, and other art. Their photorealistic content should accurately predict and indicate the film’s plot and convey its artistic appeal and the central ideological connotation of film review. However, its production and popularization are closely related to the development of the times. At the same time, to a great extent, its design form also reflects the value orientation and characteristics of the times under specific social backgrounds [10]. Film poster design changes dramatically at different times. In particular, the advent of the digital media era has posed unique requirements for film poster design. It highlights imagination, photorealistic, and visual hierarchy through advanced filmmaking techniques while considering the advertising function [11].

Today, film poster design is mainly based on visual culture, the composition of which has become the support of film poster design. Visual culture mainly includes visual text, discourse, institutions, and machines. All products based on image design can be visual text, and film posters are no exception. Visual discourse reflects the main background of visual culture, namely, its ideology, covering the design purpose and concept [12]. By comparison, the visual institution is the primary bearing mode of visual culture. A prime example is a movie theater where there are no lights in a dark hall, the lights are projected on the screen, and the audience is assigned to a specific seat set up in a specific order and hierarchy. Finally, the visual machine represents the main attributes of film viewing, including the allocation of viewing power, the composition of vision, and the betting of desire. The field of visual culture is very complex, and the critical factor in visual culture is the visual machine. The interaction between the visual machine and other factors jointly determines the audience’s viewing behavior [13].

To sum up, the film poster takes the film plot as the main content, the film’s publicity as the primary purpose, and the visual culture as the central design concept and follows the development of the times. Therefore, the origin of the film poster is closely related to the visual culture.

2) Development of film posters

As per media development, social development is divided into three critical periods: oral communication, written and printed communication, and electronic (digital) communication [14]. This division method has great practical significance. Human mentality is being reconfigured continuously with the development of the times, and the ID medium constantly evolves. In turn, the ID medium will decide the social development forms and affect human life and cultural generation [15].

Firstly, the oral communication period features a single ID mode. Hand and mouth are the most basic information communication media; the ID mode is word of mouth. Before the birth of language, sounds and symbols were the primary identifiable medium of communication, followed by Wolf Smoke (smoke as a warning signal against the war in ancient China) and other primary ID forms [16]. Since language has become the inherent ability of humans, subtle ID patterns have emerged. However, the role of ID in the common language is still minimal. Until the dawn of the written age, language was replaced by written symbols. With the continuous improvement of written symbols, humanity gradually got rid of the single way of oral communication. Then, the 1950s witnessed the birth of the printing press. The era of mechanical reproduction is ushered in, and the ID mode also changed significantly, popularizing mass communication. At this time, the printing press has become the primary ID tool. The culture of the era of print media is the culture of written notice and the culture dominated by rationality [17].

The rise and continuous S&T development diversifies ID media and enriches art forms as the primary forms of ID, such as painting, photography, television, advertising, and film. As a result, the ID mode enters the wide photorealistic world, and visual image has become one of the top ID carriers. Noticeably, the visual image is entirely different from traditional image symbols. Instead, the visual image is an updated form of ID driven by S&T. Therefore, in today’s various ID media, visual images always dominate and pivot the audience’s attention. Exquisite image-based ID has won favor in various industries as a principal means of marketing and publicity [18].

3) The impact of posters on the current film industry

The poster is a common advertising form used in the film industry for a long time. In particular, a film poster is publicity means attached to film culture, also known as a film propaganda poster. From the art perspective, film posters have gradually become independent art with technical, collection, and research value [19]. At first, film poster design was promoted and publicized through the offline exhibition. Indeed, once, it was very effective. However, in the current information-driven world, Internet-based film poster is the main form of film publicity and promotion. Meanwhile, the online poster lends well to various ID media, such as mobile phones, computers, and TV. Strengthening the online poster design and improving its publicity efficiency through state-of-the-art S&T can break the difficulty of traditional offline poster publicity. The current marketing effect of online poster production and publicity is unsatisfactory. Therefore, improving the publicity effect of film posters based on the new S&T is still the main task of the current film industry [20].

In the context of BDT, this work uses DL to generate an exclusive marketing scheme for film posters to improve their online marketing effect and sales. Ultimately, it promotes sustainable film industry development.

### Film poster analysis technology based on DL under BDT

1) BDT

**Fig 1:**
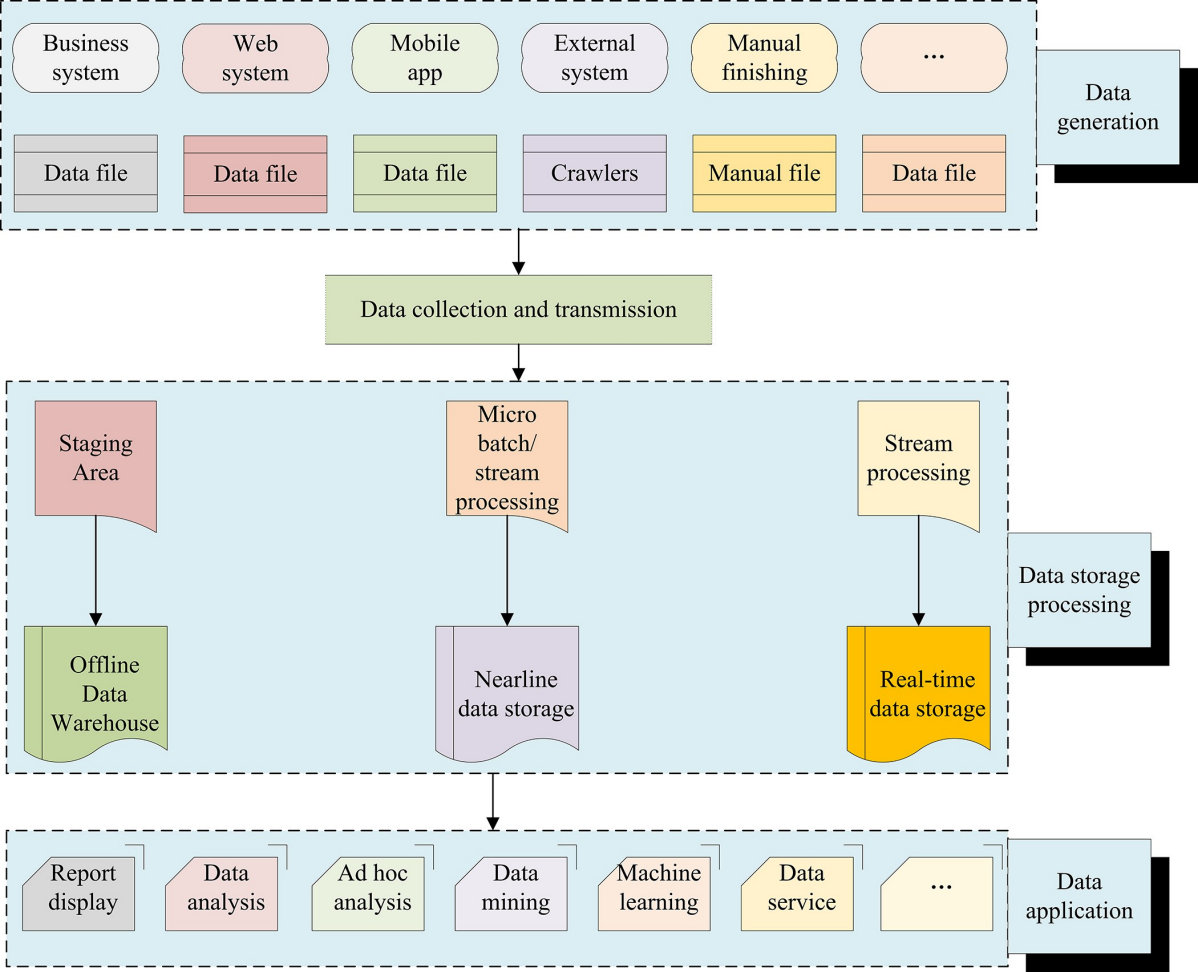
Principle of BDT.

Big Data, or massive amounts of data, specifically refer to those that cannot be retrieved, managed, processed, and sorted into helpful information to help enterprises make business decisions reasonably through mainstream software tools. The strategic significance of BDT lies not in mastering huge data information but in the industry-specified processing of these meaningful data [21]. In other words, if Big Data is compared to industry, the key to the profitability of this industry is to improve the "processing capacity" of data and realize the "value-added" of data through "processing" [22]. Fig 1 shows the main principle of BDT.

According to Fig 1, BDT is primarily composed of the data generation layer, data storage processing layer, and data application layer. The data generation layer refers to the primary source of data. Data storage and processing is the primary function of BDT, that is, to store and orientate the acquired data so that the data becomes the required form. Data application refers to the final application method after the data has undergone processing means. From a technical point of view, the relationship between Big Data and Cloud Computing is as inseparable as the two sides of a coin. A single computer cannot process Big Data, so a distributed architecture must be adopted. Its characteristic lies in the distributed data mining of massive data. However, it must rely on the distributed processing, distributed database and cloud storage, and virtualization technology of cloud computing. With the advent of the cloud era, Big Data has also attracted more and more attention [23]. Big Data is often used to describe the large amount of unstructured and semi-structured data that a company creates that would take too much time and money to download to a relational database for analysis. Big Data Analysis is often associated with Cloud Computing because real-time analysis of large data sets requires a MapReduce-like framework to distribute work to tens, hundreds, or even thousands of computers [24]. Big Data requires special techniques to efficiently process large amounts of data entered in a short period, including massively parallel processing databases, data mining, distributed file systems, distributed databases, Cloud Computing platforms, the Internet, and scalable storage systems. Therefore, Big Data has developed rapidly at the beginning of its development and has become the main form of current social information dissemination due to its powerful performance [25].

2) DL technology and film posters

As the leading ML technology, DL technology is gradually improving in the current society and has become a broadly used S&T in society. DL technology learns the inherent laws and representation levels of sample data. The information obtained in these learning processes is of great help to the interpretation of data such as text, images, and sounds. Its ultimate goal is to enable machines to have the ability to analyze and learn like humans and recognize data such as words, images, and sounds. DL is a complex ML algorithm that achieves speech and image recognition results far exceeding previous related techniques [26]. DL has achieved superior outcomes in search technology, data mining, ML, machine translation, natural language processing, multimedia learning, speech, recommendation and personalization technology, and other related fields. DL enables machines to imitate human activities such as audio-visual and thinking, solves many complex pattern recognition problems, and makes excellent progress in Artificial Intelligence-related technologies.

The DL image classification technology classifies an image through DL methods. Even though the image content may have multiple targets, it belongs to only one classification category, so the application of image classification alone is not common. However, single target recognition has the highest accuracy rate for DL algorithms. In fact, many applications will first find the target through the target detection method and then narrow the range of captured images for image classification. Image classification methods are also usually used whenever object detection can be applied. In addition, the semantic segmentation algorithm can identify each pixel in an image, that is, different from target detection. Semantic segmentation can correctly distinguish the boundary pixels of each target. In other words, semantic segmentation attains pixel-level image classification. Of course, models for such applications require much powerful computer performance and take a long time to train.

**Fig 2:**
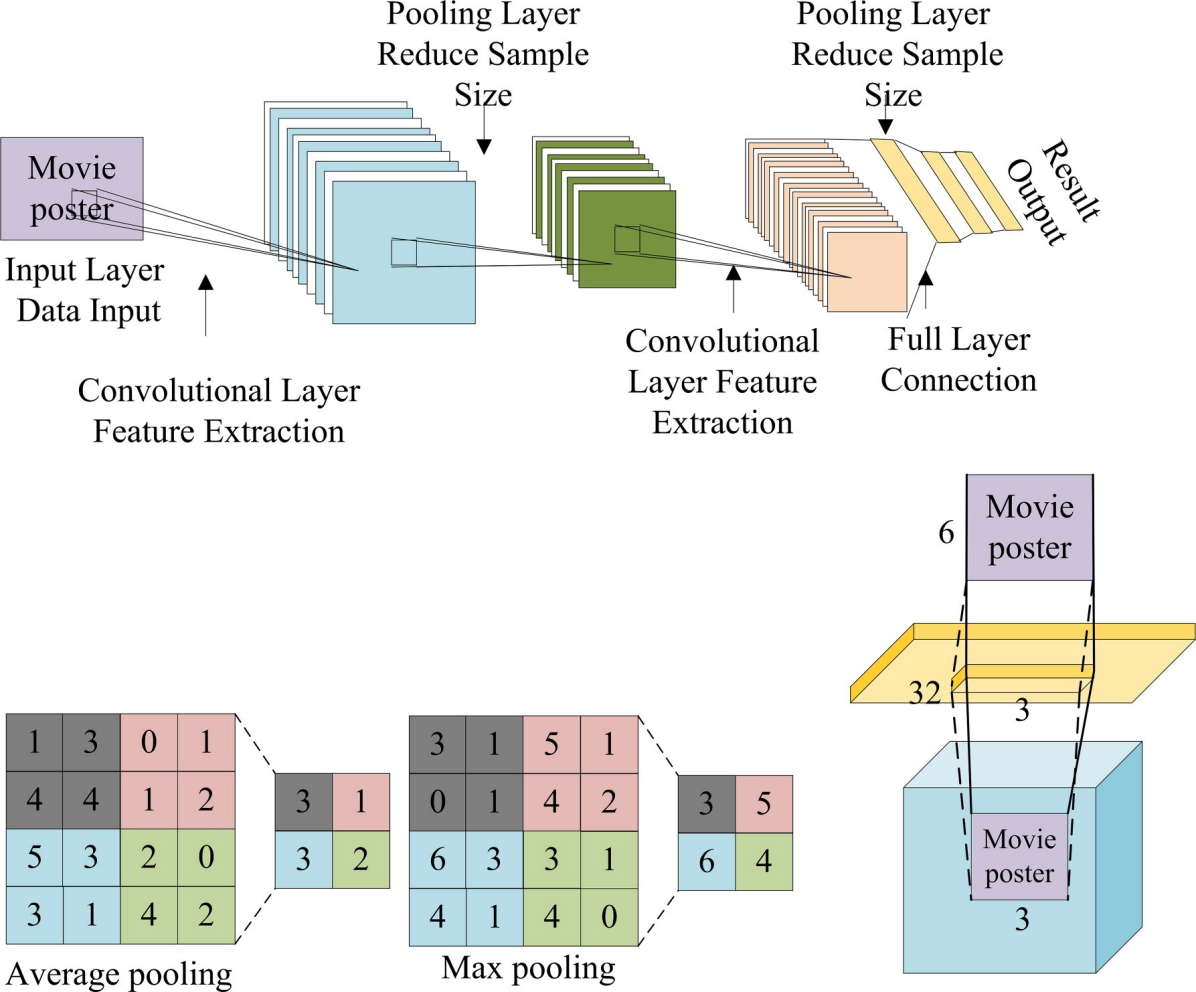
Working principle of DCNN (a: DCNN, b: The pooling operation, c: The convolution operation).

Therefore, it is a crucial innovation plan to perform image recognition through DL technology, strengthen the relevant recognition design of movie posters, and improve the marketing effect of movie posters. These measures will promote the far-reaching development of the movie industry. Deep Convolutional Neural Network (DCNN) technology is a very advantageous branch of DL technology for image recognition and analysis [27]. Fig 2 shows how the technique works of DCNNs.

The DCNN in Fig 2 includes a convolutional layer, a pooling layer, an input layer, an output layer, and a fully connected layer. Among them, the operation of Convolutional Neural Network (CNN) technology mainly includes convolution and pooling. The calculation propagation method of CNN technology principally involves forward propagation and backpropagation, which can accurately analyze images and provide analysis errors to offer data reference for model optimization to improve the calculation effect of the model. Therefore, this work analyzes the marketing plan of high-box-office movies through DCNN technology and BDT, analyzes the marketing concept of high-box-office movies, and provides a reference for movie poster marketing to comprehensively promote the film industry [28].

### DL-based film poster analysis model

**Fig 3:**
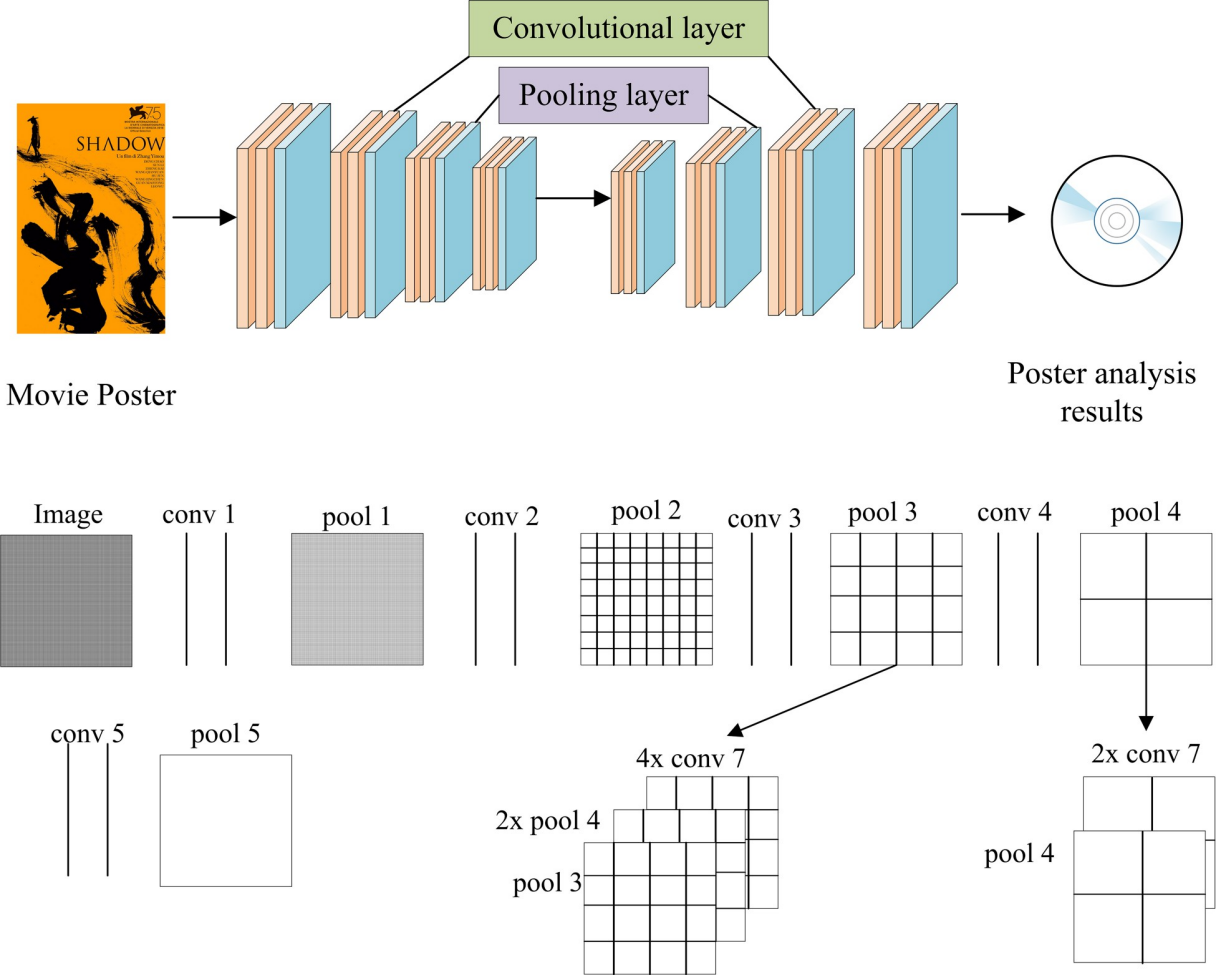
Movie poster analysis model (a: Poster analysis process; b: Poster analysis principle).

This work designs a corresponding movie poster analysis model by deep profiling of the Convolutional Neural Network (CNN) to precisely analyze movie poster recognition. Fig 3 displays the basic principle of the model designed here.

As shown in Fig 3, the typical structure of a CNN usually contains an input layer, a convolutional layer, a pooling layer, a fully connected layer, and an output layer. CNN performs feature extraction and dimensionality reduction for the input image [29]. Eq (1) describes the image feature extraction.


$$H_{i}=f(W_{i⊗}H_{i-1}+b_{i})$$ (1)

In Eq (1), ***i*** represents the network convolution level. **W** is the calculated weight, and **b** denotes the bias vector. The feature map **H**_***i***_ is obtained through the Activation Function, as presented in Eq (2).


$$H_{i}=subsampling(H_{i-1})$$ (2)

After multiple pooling and a fully connected network, the changed features are represented and classified. Eq (3) indicates the final mapping results.


$$Y(m)=P(L=l_{m}|H_{0};(W,b))$$ (3)

In Eq (3), ***m*** represents the label category index; **L** signifies the loss function; ***P*** denotes the mapping operation. The loss function can be expressed as:

$$NLL\;(W,b)=-\sum_{m=1}^{\left|Y\right|}log\;Y(m)$$ (4)

MSE(W,b)=1|Y|∑m=1|Y|(Y(m)−Y^(m))2 (5)

**Fig 4:**
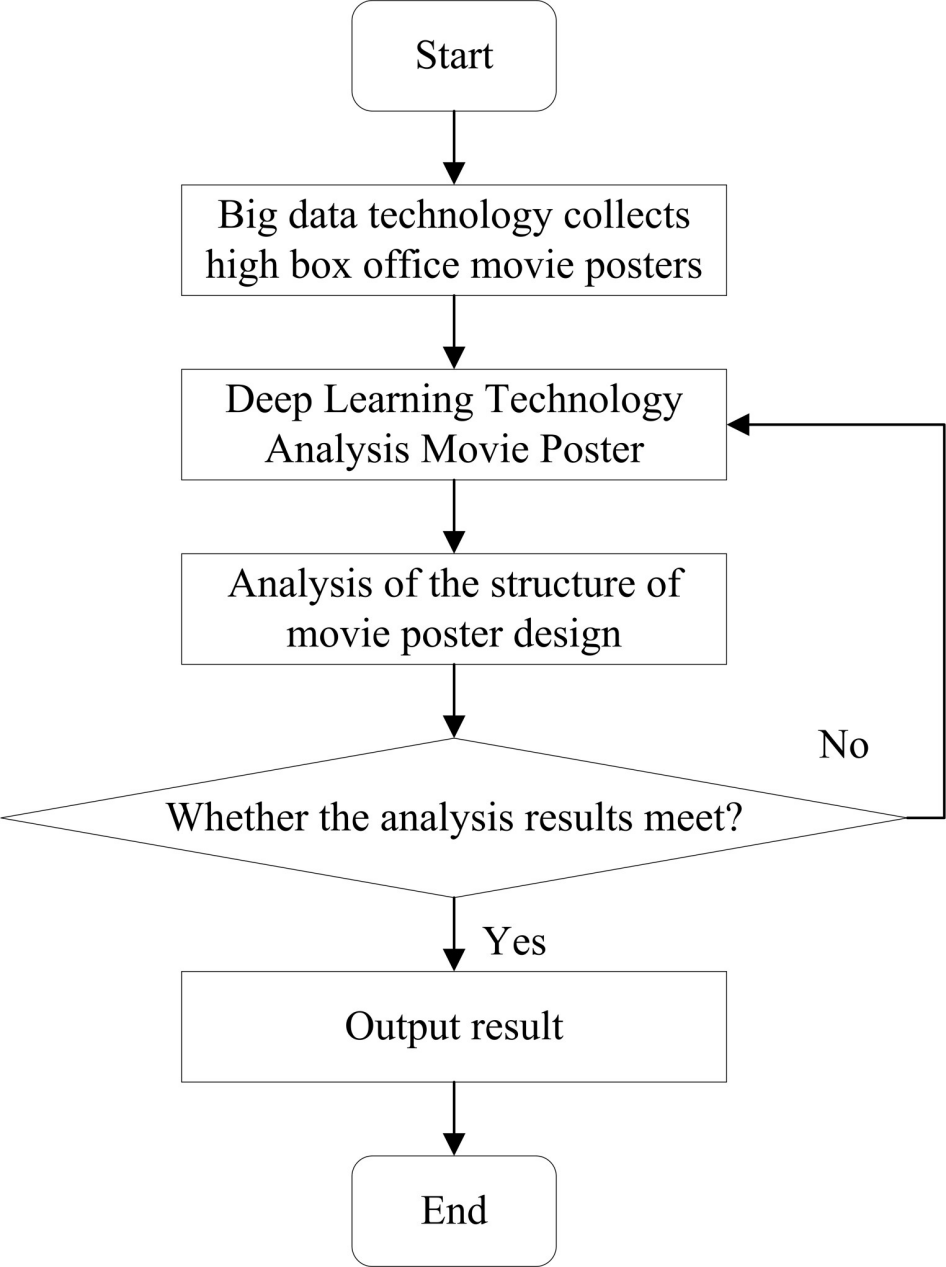
The model calculation process.

The two-norm term is usually added to the loss function to reduce the network overfitting:

$$E(W,b)=L(W,b)+\frac{\lambda }{2}W^{T}W$$ (6)

$$W_{i}=W_{i}-\eta \frac{\partial E(W,b)}{\partial W_{i}}$$ (7)

$$b_{i}=b_{i}-\eta \frac{\partial E(W,b)}{\partial b_{i}}$$ (8)

where **b**_***i***_ represents the feature paranoia of the input data; **W** signifies the feature weight of the data; ***η*** represents the learning rate. This work uses DCNN technology to identify the design principles of movie posters and improve the effect of movie poster marketing [30]. Movie poster design elements mainly include theme, style, composition, color matching, setting, and product. First, BDT is used to obtain high-box office movie posters. Then, DCNN technology is employed to analyze the six elements of high-bo office movie poster design. Finally, a reference is given for movie poster design [31]. Fig 4 reveals the main research flow of this work.

This work uses BDT and DL technology to analyze the design of movie posters. First, BDT is adopted to obtain high-box office movie posters. Then, DL technology is used to analyze the design principles of high-box office movie posters. Finally, the performance of DL technology is verified through the evaluation results. As the current advanced S&T, BDT can accurately capture the influence of movie poster design on its marketing plan through its research on movie poster design to provide a reference for the development of movie poster marketing.

### Research settings

#### Research data design

In this work, the data set is used to train the model to improve the computational performance of the model and enhance the image feature recognition ability of the model. The data set used here is the Internet Movie Database (IMDB) [32]. The information includes 5,043 movies with 28 categories and 4,906 posters, spanning 100 years and 66 countries, involving 2,399 directors and thousands of actors. Attribute items include movie title, number of reviews, rating, director, release time, release country, main actors, language, and IMDB rating. This work uses the DCNN technique to analyze the features of movie posters and compares the model training results with other models, thereby improving the analytical features of the model. The public data of this data set used here comes from the web: **http://ai.stanford.edu/~amaas/data/sentiment/**. The use of the data set is in accordance with the terms and conditions of IMDB.

#### Research environment design

Importantly, this work uses DCNN to analyze the characteristics of film posters and compares the model training results with other models to improve their performance. Anyone who has email and uses a web browser that accepts cookies can set up an account on IMDB, submit information, and participate in voting on various topics. For the demand of automatic query, most of the database can be downloaded in the format of the compressed text file and decompressed with the provided tools (usually on the command line). IMDB uses the Weighted Rank (WR) obtained by the Bayesian statistical algorithm, as shown in Eq (9).


weightedrank(WR)=(v/(v+m))×R+(m/(v+m))×C (9)

In Eq (9), **R = average for the movie(mean) = (Rating)**; **v = number of votes for the movie = (votes)**; **m = minimum votes required to be listed in the top 250**. Here, the Graphics Processing Unit hardware chooses the GTX 1080 Ti. The software environment is based on Python 2.1.1. Python is a language that represents simplistic ideas.

This work compares the DCNN model designed here with algorithm models such as K-Nearest Neighbor (KNN), Support Vector Machine (SVM), and Backpropagation (BP) to reflect the advantages of the model reported here. Moreover, the performance of the DCNN model is evaluated by the data volume of 2,000 thrillers, romantic films, drama, documentary, action films, comedy, adventure, and war movies from the six elements of movie poster design: theme, style, composition, color matching, scenery, and product.

## Analysis of film poster marketing scheme based on DL

### Model performance evaluation based on DL

**Fig 5:**
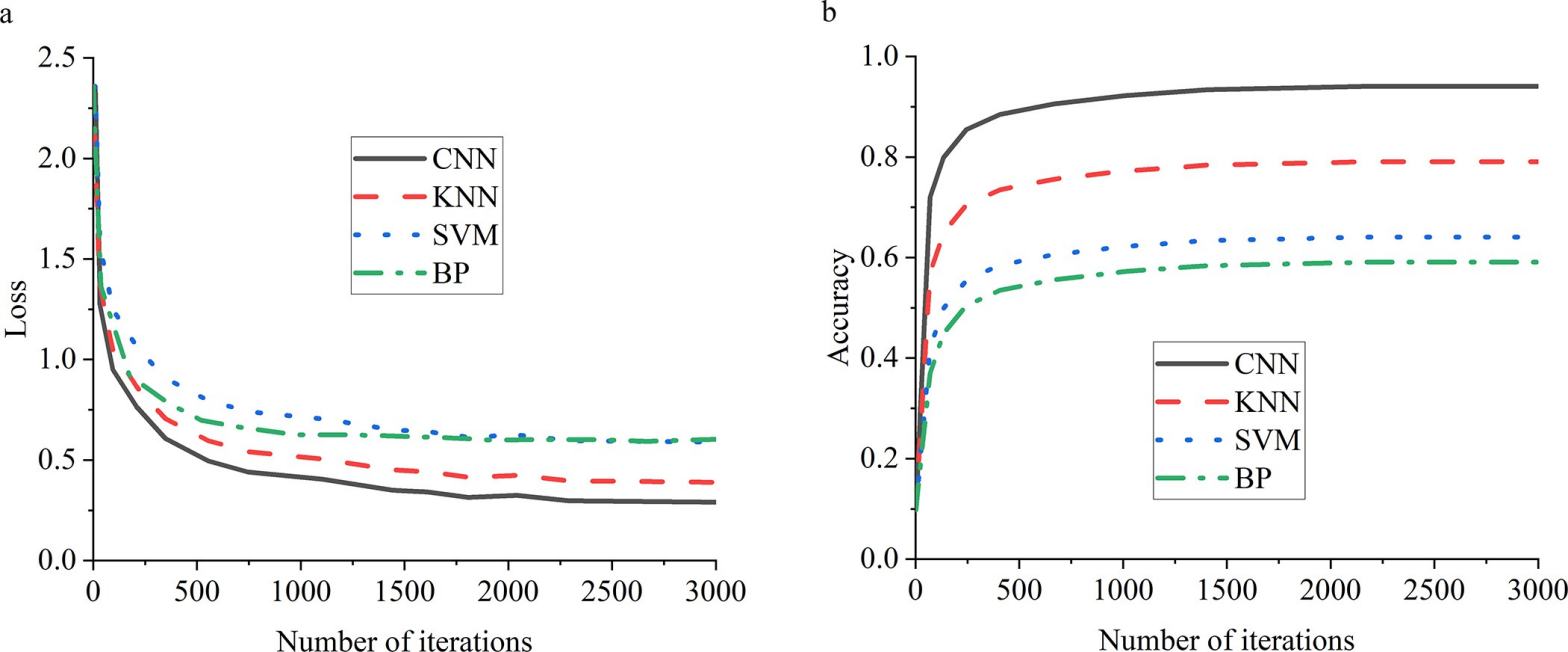
Comparison of film poster recognition performance of different models (a: learning performance, b: calculation accuracy).

DL-based image recognition is a forefront technology like the KNN, and SVM, BP. Fig 5 compares the results of image recognition performance of different models.

In Fig 5, comparing the film poster recognition performance of different algorithms reveals that the learning curve of the proposed DCNN based on BDT is the best. The learning rate of the proposed DCNN based on the BDT model is stable from the 500th iteration, and the final loss is about 0.5. Meanwhile, the accuracy of the proposed BDT-based DCNN based stabilizes from the 500th iteration, and the model accuracy reaches about 0.9.

### Effect analysis of film poster based on DL

**Fig 6:**
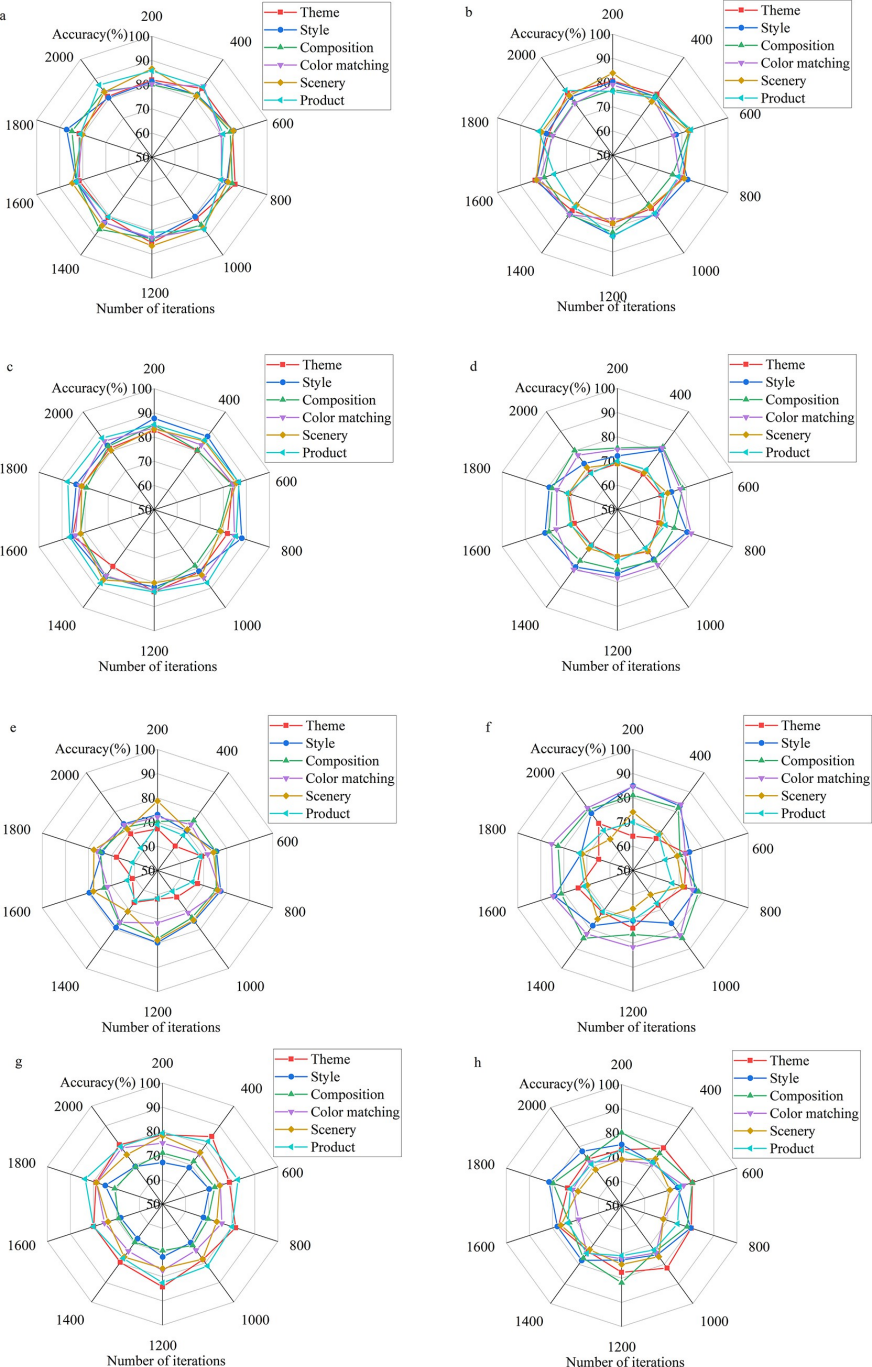
Identification and evaluation results of the film poster-oriented DCNN based on BDT (a: Horror films, b: Romantic films, c: Drama films, d: Documentary, e: Action films, f: Comedies, g: Adventure films, and h: War films).

As the main form of film publicity, the film poster effect evaluation considers many aspects according to the film classification. Here, films are mainly divided into horror, romance, drama, documentary, action, comedy, adventure, and war films. The BDT-based DCNN model reported here is trained and evaluated from the six elements of film poster design: theme, style, composition, color matching, scenery, and product. Fig 6 illustrates the evaluation results of the proposed film poster-oriented DCNN based on BDT.

In Fig 6, the DCNN model based on BDT is used to recognize and classify film posters. Regarding the style composition (theme, style, composition, color matching, scenery, and product), the DCNN model based on BDT presents a stable recognition accuracy on eight kinds of film posters. The recognition accuracy remains at 60% ~ 85%.

### Analysis of film poster marketing scheme under DL

**Fig 7:**
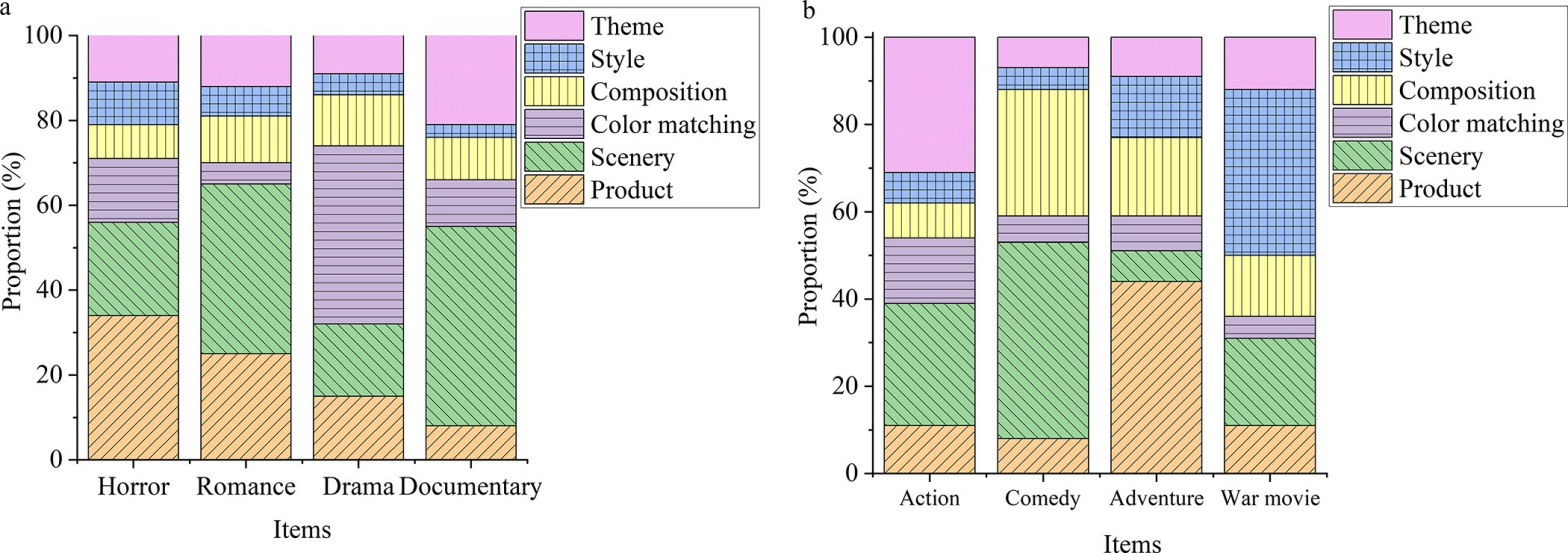
The style composition of film posters analyzed by the DCNN model based on BDT (a: Group one, b: Group two).

The proposed DCNN based on BDT can accurately identify the types of film posters. Meanwhile, it can analyze the style composition of film posters. Fig 7 evaluates the proposed BDT-based DCNN style composition analysis under 500 samples.

Obviously, according to the analysis of the BDT-based DCNN model, the style composition of film posters differs substantially from film to film. In particular, film posters pay the most attention to film product, style, and theme.

## Discussion

Artificial Intelligence has been in a tumultuous period in recent years. A proliferation of unexpected applications has been proposed, indicating that Artificial Intelligence still has a huge room for development, and the development of Artificial Intelligence technology is becoming increasingly advanced. The same data form can be analyzed by different methods to resolve different problems. For example, the face recognition technology, which is quite proficient at present, can be used for security work in national defense applications. Enterprises can use face recognition as employee access control systems. This technique can also be combined with gender and age identification to allow stores to conduct market survey analysis or with tracking technology for crowd flow analysis. DL includes many excellent technologies, among which the excellent performance of CNN technology provides a powerful driving force for the development of DL technology. Unlike regular neural networks, the neurons in each layer of a CNN are arranged in 3 dimensions: width, height, and depth. The width and height are very simple because the convolution is a two-dimensional template. In contrast, the depth in CNNs refers to the third dimension of the activated data volume rather than the depth of the entire network, which represents the number of layers of the network. DCNN is the most popular neural network model for image classification problems. The central idea of DCNN is that local understanding of the image is fundamental. It can significantly reduce parameters, shorten the time required for learning, and reduce the amount of data required to train the model. DCNN has enough weights to look at small patches of the image instead of a fully connected weight network from each pixel. Convolution is a weighted sum of pixel values of an image. It turns out that the whole process of convolution of an image with a weight matrix produces another image. This work also has excellent advantages in analyzing movie posters by designing a CNN model. The comparison of the movie poster recognition performance of different algorithms shows that the CNN technology designed here has the best learning curve. When the number of iterations is about 500 times, the learning rate of the DCNN model is basically stable; the final loss value is around 0.5. Besides, the accuracy of the model is stable when the number of iterations is about 500, and the accuracy of the model is about 0.9. In the classification and recognition of movie posters, the model reported here is relatively stable in recognition of the theme, style, composition, color matching, scenery, and products of movie posters, as well as in the recognition process of eight kinds of movie posters. Its recognition accuracy is generally between 60% and 85%. The style composition evaluation of movie posters is also analyzed through DL technology. It is found that the style composition of movie poster production in different films varies dramatically, and movie posters pay the most attention to movie products, styles, and themes. Compared with the research of Rajee and Mythili (2021) [33], this work integrates BDT and DL technology and optimizes DL technology, thus comprehensively improving the performance of movie poster analysis. Therefore, this work provides a reference for the optimization of the design method of movie posters and contributes to the development of the movie industry.

## Conclusion

The way of human information dissemination is changing with the development of technology. The film industry is an essential form of information dissemination in the art field. Meanwhile, the development of movie posters, as the basic content of movie propaganda, is an important task for the current movie industry. The current film industry presents a vague prospect. Therefore, it is necessary to study the development of the film industry through advanced science and technology to optimize its development prospects. To this end, this work discusses the design concept and methods of movie posters under BDT. Finally, a movie poster analysis model is designed based on DCNN and evaluated by experiments. A comparison of the movie poster recognition performance of different algorithms indicates that the DCNN model reported here has the best learning curve. The learning rate of the model is stable when the number of iterations is around 500, and the final loss value is around 0.5. In addition, the accuracy of the model is also stable when the number of iterations is about 500, and the accuracy of the model is about 0.9. Meanwhile, the DCNN model can recognize the theme, style, composition, color scheme, scenery, and products of movie posters. The recognition accuracy is generally 60%~85%. This work provides a reference for the development of the movie industry and promotes the optimization of the movie industry. Although this work designs an advanced DL model and makes a comprehensive evaluation of the model, the current practical application effect of the model is not reasonably evaluated. Thus, future research will make a deer analysis of the application of the model to realize the practical application and development of the model and promote the development of the film industry.

## Data statement

This work uses Deep Learning technology to realize data analysis. The address of code implementation is **https://blog.csdn.net/missyougoon/article/details/90368727**.

## Supporting information

S1 Data (ZIP)
